# Supplementary material for: Are carbon emissions trading and green financial instruments synergistic? -Comprehensive quantitative research based on content analysis
Source: PLoS One. 2024 Mar 7;19(3):e0298601. doi: 10.1371/journal.pone.0298601 (PMC10919601; doi:10.1371/journal.pone.0298601)
Supplement: S1 Data — (ZIP) [file pone.0298601.s001.zip › Data/地级市Z_F工作报告.zip!/─┌├╔╣┼/╢⌡╢√╢α╦╣╩╨/└┤╫╘╣1⁄2╓┌║┼í╛┬φ┐╦╩2╛▌═°í┐_2014.docx]

各位代表：

　　现在，我代表市人民政府向大会作工作报告，请予审议，并请市政协委员和列席会议的同志们提出意见。

一、2013年工作回顾

　　过去的一年，面对复杂严峻的经济下行态势，市人民政府认真贯彻落实中央的方针政策和自治区“8337”发展思路，在市委的正确领导下，在市人大常委会和市政协的大力支持下，牢牢把握稳中求进的工作总基调，团结带领全市各族干部群众，攻坚克难，扎实工作，全面完成了各项目标任务，经济社会发展实现稳中有进、稳中向好。

　　（一）整体经济保持平稳增长。全市预计完成地区生产总值3965 亿元，增长9.5%；公共财政预算收入440 亿元，增长17.2%；固定资产投资2996 亿元，增长16.5%；社会消费品零售总额551.5 亿元，增长10%。农牧业生产再获丰收，粮食产量达到31 亿斤，牧业年度牲畜存栏1223 万头（只），一产实现增加值95 亿元，增长3%。工业经济较快增长，完成投资2122 亿元，增长38%；规模以上工业增加值1897 亿元，增长12.7%；工业用电量增长11.4%。新增超百亿元园区2 个、企业1 户。园区实现销售收入2100亿元，增长27.9%。现代服务业稳步发展，金融机构存贷款余额分别达到2239 亿元和2362 亿元，增长5.5%和7.3%；公路、铁路货运量增长14%和12%；外贸进出口总额增长1.6倍；接待游客人数和旅游收入增长12.2%和22.1%。第三产业实现增加值1488 亿元，增长7%。县域经济和非公有制经济实力增强，分别占全市经济总量的78%和70%。

　　（二）产业转型升级成效明显。编制了清洁能源、现代煤化工、装备制造、铝循环等重点产业发展规划。开复工亿元以上重大项目168 项，其中非煤产业项目108 项。中海油、北控、河北建投、新蒙200亿立方米煤制气和中电投80 万吨烯烃、伊泰200 万吨煤液化等24 个重大项目已经国家批复或同意开展前期工作，中天合创煤制甲醇、伊泰精细化学品、满世大化肥等51 个重大项目开工建设，中煤大化肥、奇瑞汽车、京东方半导体显示器件、新兴重工压力容器等30 个重点项目建成投产，新增电力装机202 万千瓦、煤化工产能390 万吨、汽车产量6.9 万辆。非煤产业完成投资1700 亿元，占工业总投资的80%；完成增加值652 亿元，占规模以上工业增加值的34.4%，比上年提高了4个百分点。

　　（三）民生保障水平持续提高。全年完成民生投入422.7 亿元，占公共财政预算支出的81.5%。市委、市政府年初确定的9 个方面惠民实事全部落实。新增城镇就业3.3万人，城镇登记失业率控制在2.7%以内。企业退休人员养老金由每人每月2265元提高到2433 元，城乡居民养老金分别达到每人每月550 元和310 元，城乡低保标准由每人每月460 元和340 元提高到480 元和390 元。将20 种重大疾病纳入新农合保障范围，城乡居民医疗保险人均筹资标准分别达到563 元和574 元，报销封顶线提高到20 万元。覆盖全市的社会保障体系已基本建立。全面实施营养早餐工程，14.5 万名小学生和幼儿受益。给农村牧区教师每人每年补助1 万元。回购和新建保障性住房10770 套，改造沿黄渗漏区等农村牧区危旧房4750 户。改建通村公路612 公里，解决了8000 户农牧民用电、5.8 万人安全饮水问题。2.6万贫困人口稳定脱贫。生态移民生活补贴发放年限由5 年延长至8 年。城镇居民人均可支配收入和农牧民人均纯收入分别增长9%和11%，达到36123 元和12672元。

　　（四）城乡统筹发展步伐加快。城市服务功能进一步完善，中心城区基础设施加速融合对接，东康快速路改扩建主体工程完工，机场高速公路、市体育中心等重点建设项目进展顺利。全市完成市政基础设施和园林绿化投资106亿元，新增城市绿地2600万平方米。旗府所在地城镇建设有序推进，启动了7个重点苏木乡镇建设工程。新农村新牧区建设力度加大，建成中心居民点9个。鄂前旗城乡统筹、杭锦旗扶贫开发、达旗县域经济发展示范区建设步伐加快。

　　（五）生态和基础设施建设力度加大。开展了大规模的重点区域绿化和义务植树活动，完成林业生态建设166.2万亩、退牧还草370万亩、水土流失治理180.9 万亩。我市被评为“全国绿化模范城市”。节能减排力度加大，完成了自治区下达的减排任务，万元GDP能耗下降3.43%。实施重大交通项目33项，准兴重载高速、察汗淖至敖镇一级等公路和南部、巴准等铁路项目建成通车，机场新航站楼和包西铁路东胜西客运站投入运营，新增高等级公路370公里、铁路296公里。建设高标准黄河堤防115公里，哈头才当、蒙西等供水工程建成投用，新增城市和工业供水量3000万立方米。新建110千伏以上输变电线路169公里，改造农网线路1.2万公里，实现农村牧区户户通电。

　　（六）社会事业发展取得长足进步。加强师资队伍建设，基础教育整体发展水平进入全区前列。新建改扩建幼儿园40所、中小学9所。内大鄂尔多斯学院二期工程、生态环境职业学院新校区建成投用。新建国家和自治区重点实验室2 个、院士工作站3 个、工程技术研究中心2个。市高新技术产业园区被列为国家清洁能源国际创新园。举办了内蒙古“草原英才”高层次人才合作交流会，引进合作机构35 个、海内外高层次人才162人。文化惠民工程深入推进，我市成为全国首批国家公共文化服务体系示范区。大力开展全民健身运动，新建社区健身活动中心12个。优化医疗资源配置，推行旗乡村卫生服务一体化管理模式，市人民医院、第三人民医院、妇幼保健院建成投用，市人民医院与市中心医院、市中医院与康巴什医院实现一体化运营，建成标准化卫生室380个。加强人口和计划生育工作，人口出生率控制在10.35‰以内。

　　（七）社会管理水平不断提升。加强民间借贷、房地产和政府性债务领域风险防控，通过化解政府性债务、打击非法集资、债权债务抵顶、回购团购商品房等措施，妥善化解了一批民间借贷案件，房地产市场总体平稳。信访、维稳和安全生产形势稳定，信访总量下降20%，刑事和治安案件下降9.5%和11.6%，安全生产事故起数和死亡人数下降4.6%和7.9%，我市被评为“全国社会管理综合治理优秀市”。食品安全监管体系进一步完善，成为国家食品生产质量安全示范城市试点。精神文明和民主法制建设深入推进，我市城市文明程度指数测评位列全国地级文明城市第五位。国防预备役、国防动员能力建设不断加强。民族宗教工作取得新成绩。

　　（八）政府自身建设进一步加强。坚持依法行政，自觉接受人大、政协和社会各界的监督，人大代表建议和政协委员提案全部办结。取消和下放行政审批权104项、执法权28项、收费项目7项。认真贯彻落实中央“八项规定”，切实转变工作作风，政府会议、文件和公务接待活动分别减少24%、36%和48%。集中开展了党政机关公务用车和办公用房专项治理，清理超标公务用车2000余辆、超标办公用房10万多平方米。大力压缩一般性支出，公用经费和常规性支出压减了20%和50%。积极推进政务公开，加强行政监察和审计监督，制定出台重大项目稽查、政府投资项目审计监督、矿产资源交易等一系列管理办法，完善了公共资源交易运行机制，廉政建设和反腐败工作取得新成效。

　　各位代表，过去一年的成绩来之不易。这是自治区党委、政府和市委正确领导的结果，是市人大、市政协和社会各界支持监督、共同努力的结果，是全市人民同心同德、团结奋斗的结果。在此，我代表市人民政府，向全市各族干部群众和所有关心、支持、参与鄂尔多斯建设发展的社会各界人士，表示衷心的感谢并致以崇高的敬意！

　　受宏观经济形势影响，我市经济社会发展中也遇到了不少困难和问题。主要表现在：主要经济指标增速回落，部分企业生产经营困难，财政收支矛盾突出；主导产业单一化问题凸显，企业创新能力不强，资源环境和要素制约进一步加大，发展的质量和效益有待提升；城乡之间、区域之间、经济和社会之间发展不够协调，农村牧区生产生活条件还比较落后；政府性债务规模较大，民间借贷和房地产领域还有潜在风险，资源开发、征地拆迁、回迁安置、农民工工资拖欠等引发的矛盾纠纷增多，社会管理还存在薄弱环节；政府职能转变还不够到位，工作作风和办事效率仍需改进提高；铺张浪费、吃拿卡要、违法违纪等现象在一些地区、部门和单位仍然存在。对此，我们将高度重视，采取有力措施，切实加以解决。

　　二、2014年主要工作任务

　　2014年，是贯彻落实党的十八届三中全会精神、全面深化改革的第一年，也是深入贯彻落实自治区“8337”发展思路、实现“十二五”奋斗目标的关键一年。总的看，今年我市发展既面临着一系列有利条件和难得机遇，也面临着诸多压力和挑战。世界经济延续缓慢复苏态势，国内经济温和回暖，市场预期有所好转，特别是国家全面深化改革和推进新型城镇化建设，将进一步激发经济社会发展的内生动力和活力。国家治理大气污染、发达地区加速产业转移、自治区建设“五大基地”等机遇，为我市清洁能源和现代煤化工等优势产业发展提供了广阔空间。但是，我们面临的国内外经济形势依然复杂严峻，我市已进入经济增长换挡期、结构调整阵痛期和财政收支矛盾凸显期相互叠加阶段。我们既要看到有利条件和积极因素，又要充分考虑经济形势的不确定性和困难情况，坚定信心，振奋精神，迎难而上，全力推动经济社会持续稳定健康发展。

　　今年政府工作的总体思路是：深入贯彻党的十八大、十八届三中全会精神和自治区“8337”发展思路，全面落实中央和自治区经济工作会议精神，坚持稳中求进的工作总基调，把改革创新贯穿于经济社会发展各个领域和环节，着力保持经济稳定增长，着力加快产业转型升级，着力推进城乡统筹发展，着力推动重点领域改革，着力保障和改善民生，切实提高经济增长质量和效益，提高人民生活质量和水平，实现经济持续健康发展和社会和谐稳定。

　　经济社会发展的主要预期目标是：地区生产总值增长9%左右，突破4000亿元，达到4300亿元；公共财政预算收入增长2%，完成450亿元；全社会固定资产投资增长13%，突破3000亿元，完成3390亿元；规模以上工业增加值增长12%，突破2000亿元，达到2100亿元；社会消费品零售总额增长9.5%，达到600亿元；城镇居民人均可支配收入和农牧民人均纯收入分别增长9%和10%，达到39370元和13940元。完成自治区下达的节能减排目标任务。居民消费价格指数涨幅控制在3%以内。

　　今年，市政府将重点抓好六个方面工作：

　　（一）调整优化产业结构，推进“五大基地”建设，促进经济持续健康发展。立足我市产业发展实际，按照自治区“8337”发展思路要求，全力打造国家清洁能源输出基地、国家新型煤化工生产示范基地、全区铝循环和装备制造业基地、全区绿色农畜产品生产加工基地和旅游观光、休闲度假基地，推动产业转型升级，促进经济持续健康发展。

　　重点发展清洁能源和现代煤化工产业。稳定煤炭生产，着力提高清洁利用水平，全面推广煤炭洗选、低温脱水等洁净利用技术，煤炭洗选率提高到85%以上。实施煤电基地开发规划，推进蒙泰、君正、魏家峁等电厂建设，新增电力装机120万千瓦。加大天然气勘探开发力度，天然气产量达到280亿立方米。积极发展风能、太阳能等新能源产业。大力发展煤基精深加工产业，加快煤制油、煤制气等清洁能源项目建设，延伸煤制甲醇、煤制烯烃等产业链条，抓好神华煤制油第二、三条生产线、伊泰200万吨煤液化、中天合创煤制烯烃、中电投烯烃、满世大化肥和中海油、北控、河北建投、新蒙煤制气等重大项目建设，确保久泰烯烃、汇能煤制气等项目建成投产，推动煤化工产业向规模化、基地化、集群化方向发展，新增煤化工产能390万吨。加快电力、化工产品外送通道建设，推进蒙西至天津南、上海庙至山东特高压输电线路前期工作，开工建设陕京四线天然气输送管道工程。

　　培育发展新兴产业。提高富铝煤炭资源综合利用水平，积极发展粉煤灰提取氧化铝及铝产品精深加工业，加快鑫恒、蒙西、神华、大唐、中铝等项目建设步伐，构筑煤电铝一体化循环产业链。大力发展以汽车为主的装备制造业，支持奇瑞、华泰、精功恒信等企业做大做强，继续引进零部件配套生产企业，年内生产销售汽车15万辆、发动机35万台、变速器15万台，零部件配套生产企业达到40家。加快发展煤炭机械、化工设备等制造业，培育新的产业优势。积极发展电子信息产业，争取京东方半导体显示器件项目实现批量化生产，加快罗克佳华物联网应用基地、富士康精密仪器、云计算产业园数据中心等项目建设。改造提升建材、陶瓷等传统产业，推进产品高端化、品牌化、系列化发展。

　　做大做强农畜产品加工业。加快建设绿色农畜产品生产加工输出基地，培育发展肉羊、肉牛、生猪、家禽、瓜果蔬菜和林沙产业，新建改扩建集约化养殖园区27个，新建现代农业基地25万亩，发展瓜果蔬菜基地31万亩。大力扶持绒纺产业发展，实施优质绒山羊保护繁育工程，提升绒纺产业创新能力，完成轻纺工业园搬迁工作。加强与深圳海王、台湾青溪总会等企业合作，建设沙棘、螺旋藻等深加工项目。进一步完善农畜产品流通和社会化服务体系，加快新型农牧业经营体制和方式改革，鼓励发展家庭农牧场、专业大户、农牧民合作社、产业化龙头企业等新型主体，新建农牧民专业合作社40个，新增市级以上龙头企业34家，启动建设3处大型农畜产品仓储物流园区，完成37个基层农牧业公共服务综合站建设任务。

　　加快发展现代服务业。集中打造空港园区，加快引进一批现代物流、电子信息、总部经济和航空商务项目，推进综合保税区建设。积极主动与天津、秦皇岛等港口对接，支持本土企业与国内大型物流集团合作，加快札萨克、大塔等4个大型煤炭物流园区建设。继续引进银行、证券、保险等金融机构，积极发展互联网金融，增设期货交割库，争取光大、乌海银行挂牌运营。扩大银行信贷投放规模，加大中小企业集合债券、市政债券发行力度，支持我市企业到全国中小企业股份转让系统挂牌，争取满世、建元、棋盘井矿业等企业上市，年内融资规模超过500亿元。大力发展文化旅游、休闲娱乐产业，抓好蒙古源流、乌审旗“一河三园”等文化旅游景区建设，规划完善精品旅游线路，打造体现草原文化、独具地区特色的旅游观光和避暑休闲度假基地。积极发展电子商务、家政服务、健康养老等新型服务业态。第三产业实现增加值1575亿元，增长6.5%。

　　（二）推进以人为核心的城镇化建设，改善农村牧区基础条件，提高城乡统筹发展水平。

　　积极稳妥推进新型城镇化。加强与周边城市融合对接，推进呼包鄂城市群建设。科学确定中心城区开发强度，合理控制建设规模，年内不新建单纯商品房开发项目，加快消化存量商品房。进一步完善城市基础设施、商业设施和公共服务体系，推进智慧城市建设，营造宜居宜业城市环境，加快集聚人气商气。推进各组团基础设施配套衔接，实现城市公共交通、供水、供气、供热以及公共文化等设施共建共享、一体化运营。实施东康快速路沿线、阿布亥沟、红海子湿地等重点绿化工程，建设国家园林城市。统筹抓好中小城镇规划、建设和管理，进一步优化城镇空间布局，继续推进重点中小城镇基础设施和公共服务工程建设，打造规模适度、功能完善、环境优美、宜居宜业的特色城镇。推进城镇和园区融合互动发展，培育各具特色的产业体系，提高城镇产业支撑水平。以稳定职业和稳定住所为基本条件，放宽外来人口落户条件，建立城乡一体、自由流动的户籍管理制度，有序推进农牧业转移人口市民化，享受城市公共服务和社会保障。继续推进鄂前旗城乡统筹、杭锦旗扶贫开发、达旗县域经济发展示范区建设。

　　改善农村牧区生产生活条件。进一步优化村庄布局，完善中心居民点配套设施，提高自来水、太阳能、沼气、通讯网络普及率，打造现代化新型村庄。集中整治城乡结合部、交通沿线和农村牧区环境脏乱差问题，建设美丽乡村。统筹推进农村牧区农田水利、道路交通、电网通讯等基础设施建设，加大农村牧区危旧房改造力度，认真研究解决禁止开发区不具备搬迁条件农牧民的生产生活困难。完善扶贫联系包扶机制，加大金融扶贫力度，整合扶贫开发项目和资金，实施精准扶贫，实现2.6万贫困人口稳定脱贫。

　　加强生态文明建设。严格落实农村牧区“三区”发展规划和禁牧、休牧、划区轮牧政策，加强林木管护和森林、草原防火工作，巩固生态建设成果。实施好京津风沙源治理二期、十大孔兑综合治理等重点生态工程，加快重点区域绿化，完成造林130万亩、草原建设610万亩、水土流失治理180万亩。推进国家森林城市建设。加强水源地保护，确保水资源供应和饮用水安全。严禁工业生产抽采地下水，鼓励中水回用。创建国家环保模范城市，严格执行环保“三同时”制度，实现市、旗、园区和企业在线监管系统全覆盖。增强基层环保执法力量，建立环保执法联席会议和社会监督制度。落实大气污染防治和主要污染物减排措施，循环利用废水、废气、废渣，年内所有化工类园区建成污水和废渣处理设施。突出抓好以土地复垦为重点的矿区环境综合治理，建设和谐矿区。

　　提升基础设施承载能力。推进荣乌高速棋盘井至乌海、红庆河煤矿至杭锦旗锦泰化工园区、阿门其日格至小壕图、通史至靖边等公路和呼准鄂、准朔、蒙西至华中等重点铁路项目建设，畅通省、区际出口通道，新增高等级公路325公里，新建改建通村公路500公里，新增铁路里程200公里。提高机场运营管理水平和服务质量，优化国内航线航班，合理开通国际航线，积极做好通勤机场前期工作。加快重点城镇、园区供水工程建设，完成水权转换二期工程，新增供水能力6000万立方米。加快电网改造升级步伐，新建110千伏输变电线路720公里、220千伏输变电线路630公里。

　　（三）着力保障和改善民生，统筹推进社会事业发展，不断增进人民群众福祉。

　　持续加大民生改善力度。认真实施扶贫攻坚、创业就业、百姓安居、平安创建工程，进一步完善现行惠民政策，巩固民生成果。今年的民生工作要坚持“雪中送炭”，重点向弱势困难群体和农村牧区倾斜，集中办好十项惠民实事。一是新增城镇就业3.1万人，其中安置鄂尔多斯籍高校毕业生1万人，城镇登记失业率控制在3.5%以内；发放小额贷款2.5亿元，扶持6000人自主创业；建设市公共实训基地；完成就业培训3.2万人次；开通鄂尔多斯市家政服务网，免费培训家政服务人员1500名；充分保障农民工权益；建设大学生创业园刀遣扇⌒陆ā⒒毓骸⒏脑斓确绞剑略鲇锥?1所，集中解决公办幼儿园“入园难”问题。三是改造农村牧区危旧房1万户，其中沿黄渗漏区危房2800户；改造城市棚户区8000户。四是解决3.5万人安全饮水问题。五是优化公交线路和站点设置，实现中心城区公共交通一体化运营。六是参加医保患者在全市定点医疗机构住院治疗费用实现即时结报。七是在落实国家和自治区提标政策的基础上，将独生子女伤残、死亡家庭扶助标准每人每月提高100元；对贫困妇女乳腺癌、宫颈癌患者一次性提供1万元救助资金。八是对社会投资新建、改扩建养老机构给予建设补贴和床位补贴；对年满60周岁城乡低保户、“三无人员”、优抚对象等重点保障人群给予每人每年600元居家养老补贴。九是将城乡低保标准分别由每人每月480元和390元提高到495元和402元，五保集中供养标准由每人每年7840元提高到8000元；为肢体三级残疾人每人每年发放补贴2000元；建设市残疾人创业园。十是实施食品质量安全追溯体系建设工程。

　　推进社会事业改革发展。落实考试招生改革制度，试行公办学校校长、教师交流轮岗制度，不设重点学校、重点班，破解择校难题，探索建立义务教育均衡发展的政策措施。实施基础教育标准化建设工程，全面完成中小学和幼儿园续建任务。开展早期幼儿教育试点工作，加快发展高等教育、职业教育、民族教育，促进教育事业全面协调发展。推进公立医院改革，鼓励社会资本办医和医师多点执业。利用信息化手段，实现优质医疗资源共享。完善重特大疾病医疗保险和救助制度，健全突发公共卫生事件应急处理、疾病预防控制体系。加快蒙中医药事业发展。加强公共文化服务设施建设，大力开展群众性文体活动，巩固国家公共文化服务体系示范区创建成果。做好第十届全国少数民族传统体育运动会筹备工作。加强人口和计划生育工作，提高出生人口素质，启动实施一方是独生子女的夫妇可生育两个孩子的政策。保障妇女儿童权益，抓好城乡老龄事业发展。稳步扩大社会保险覆盖面，完成国家社会保险标准化试点建设任务。

　　（四）全面推进改革创新，突破体制机制制约，推动地区经济更有效率、更加公平、更可持续发展。

　　加快重点领域改革步伐。认真落实中央、自治区全面深化改革的各项政策措施，加快资源型城市创新发展试点建设。加强和改进预算管理，建立跨年度预算平衡机制，合理界定市旗两级事权划分和财政支出责任，实行全口径预算管理，稳步推进财政预决算、部门预决算和“三公”经费公开。在公共事务性领域推行政府购买服务。强化税收征管，挖掘税收潜力，清理规范税收优惠政策，推进依法治税。加快市直国有企业整合重组，鼓励地方民营企业参股国有企业，进一步增强国有企业活力，提高国有资本收益。完善资源配置政策，严格按转化项目、按市场价格配置煤炭资源，探索建立已配置资源但未兑现转化项目退出机制。加快推进资源资本化改革，利用矿产资源通过资本市场直接融资。建立城乡统一的建设用地市场，组建市旗乡三级土地流转平台，允许农村牧区集体经营性建设用地出让、租赁、入股，增加农牧民财产性收入。加大金融改革创新力度，开展土地信托流转试点工作，鼓励民间资本参与地方金融企业增资扩股，支持有条件的本土企业发起设立民营银行。实施跨区域水权转换和水沙置换工程。积极争取与能源输入地环境容量指标置换，鼓励企业参与排放权交易，推行环境污染第三方治理。扩大电力多边、双边交易，促进市域电力资源优化配置。

　　发展壮大非公有制经济。坚持权利、机会、规则平等，按照“非禁即入”原则，推行“负面清单”管理模式，清理、调整前置审批事项，取消最低注册资本限制，促进非公有制企业公平进入基础设施、市政公用事业、社会事业和金融服务等领域。扶持本土民营企业做大做强，鼓励本土民营企业与引进企业相互参股、嫁接合作，实现资源、资本、技术、人才、管理优势互补。引导非公有制企业运用高新技术改造提升传统产业，发展非资源型产业。支持国有资本、集体资本、非公有资本交叉持股、相互融合，发展混合所有制经济。大力实施服务企业工程，全面落实扶持企业发展的各项政策措施，切实帮助企业解决信贷融资、市场开拓、项目建设要素制约等困难和问题，促进企业稳定生产，全力扶持困难企业度过难关。

　　提升对外开放和科技创新水平。重点抓好园区招商和产业链招商，积极引进国内外高端产业、先进技术和资金，主动承接发达地区产业转移，引进国内（区外）资金640亿元。加强区域合作，在清洁能源供需领域与能源主要消费区建立互惠共赢的合作关系。加大科技投入力度，加强与中科院、清华大学等科研院所合作，完成清华低碳谷一期建设任务。做好国家级高新技术产业园区申报工作，加快软件园、孵化器建设。深入实施“人才鄂尔多斯”战略，统筹培养和引进各类人才，提高人才工作水平。

　　（五）加强和创新社会管理，落实风险防控措施，确保社会和谐稳定。

　　创新社会管理机制。推进国家社会管理创新试点城市建设，加快构建社会管理主体多元化格局。推行网上受理信访事项，落实涉法涉诉信访案件依法终结制度。加大资源开发、环境污染、回迁安置、农民工工资兑付等领域矛盾纠纷排查调处力度，积极化解信访积案。深入推进平安鄂尔多斯建设，完善立体化治安防控体系，提升治安防控信息化和社会化水平。严厉打击各类刑事犯罪，切实提高合成作战能力。加强社区矫正工作。强化食品药品安全监管，严格标准，严厉处罚，严肃问责，确保人民群众饮食用药安全。加强社会诚信体系建设，建立信用“黑名单”和失信惩戒制度，严厉打击隐匿资产、恶意逃债、有钱不还等赖账行为，塑造诚信鄂尔多斯形象。完善应急体系，加强应急队伍建设和救援演练，提高处置突发事件和自然灾害的能力。积极稳妥解决民间借贷纠纷，最大限度保障债权人合法权益。高度重视网络舆情，正面引导社会舆论。加强国防宣传教育，强化国防动员能力建设。

　　严防政府性债务风险。切实增强政府性债务风险防控意识和能力，进一步摸清底数、界定性质、厘清范围，通过预算安排、压缩支出、资产拍卖、债权相抵等措施，积极筹措资金，全力做好政府性债务清理化解工作，确保完成三年化债任务。政府偿债资金优先用于偿还农民工工资和化解民间借贷案件。坚持“化旧”与“控新”并重，健全政府性债务管理制度，严格举债审批程序，落实偿债责任，建立偿债准备金制度，把政府性债务分门别类纳入全口径预算管理。规范政府融资行为，清理整合政府性投融资平台。加大考核问责力度，树立“还债也是政绩”的理念，将化解政府性债务工作纳入实绩考核重点，严厉查处违规举债和化解债务不力的责任人。

　　加强安全生产监管。健全完善安全生产责任体系，落实“党政同责、一岗双责”制度，强化企业主体责任。加大安全生产投入力度，加强企业员工安全培训，从源头上杜绝安全生产事故发生。加大执法检查力度，充实安全生产专业监管力量，强化对煤矿、危化企业、建筑、消防、道路交通、油气管道、民爆行业等重点领域的安全监管。严格事故查处和责任追究，建立安全生产检查连带责任追究制。高度重视消防安全，提高消防装备水平，年内所有园区建成消防队站。做好防凌防汛、城镇防洪防涝工作，保障人民群众生命财产安全。

　　（六）转变政府职能，改进工作作风，努力建设法治政府和服务型政府。

　　优化发展环境。继续简政放权，削减下放行政审批事项和收费项目。对列入自治区“8337”发展思路重点规划的项目，除须经国家审批的外，一律改为备案制。全面推行集中办理、网上审批和满意度测评制度，优化审批流程，减少审批环节，提高审批效率。坚持依法行政，加强法治政府建设，自觉接受人大、政协和社会各界监督，制定法治鄂尔多斯建设实施纲要，加强法制宣传教育，改善地区法治环境。整合行政执法资源，规范行政执法行为。推进政府机构改革和事业单位分类改革。

　　改进工作作风。严格落实中央、自治区和市委改进工作作风、密切联系群众的各项规定，扎实开展党的群众路线教育实践活动，突出整治“四风”问题。继续精简会议和文件，严控各类会展、论坛、节庆和检查评比活动，绝不搞华而不实、劳民伤财、急功近利的形象工程。强化行政监察和政务督查，加大行政问责力度，坚决查处庸懒散奢、吃拿卡要、效率低下、不负责任、执行不力、弄虚作假等行为，确保政令畅通。深入开展行风治理，集中整治学校、医疗机构、交管、运管、煤炭稽查等领域乱收费、乱罚款等损害群众利益的行为。加强公务员队伍建设，强化公仆意识、服务意识、责任意识，建设一支作风优良、纪律严明的公务员队伍。

　　厉行勤俭节约。全面落实《党政机关厉行节约反对浪费条例》，牢固树立过紧日子的思想，坚决杜绝大手大脚、铺张浪费。调整优化财政支出结构，按照“四保两压”原则，优先保运转、保民生、保化债、保重点续建项目，进一步压缩“三公”经费和政府投资项目支出规模，大幅压缩或取消历年安排的常规性项目支出。除民生项目外，原则上不开工新建政府投资项目。从严控制差旅费等行政经费支出，一律不参加没有实质内容的招商引资和外出考察培训等活动。不折不扣地贯彻落实国务院“约法三章”要求，政府性楼堂馆所一律不得新建，财政供养人员只减不增，公费接待、公费出国、公费购车只减不增。加强对公务员队伍勤俭节约的监督约束，带动全社会形成节俭之风。

　　强化廉政建设。完善市旗两级公共资源交易平台建设，严格执行政府投资项目社会公开和招投标制度，将所有公共资源纳入平台集中交易。严控工程建设规模和标准，坚决杜绝超预算、超标准建设行为，推行项目建设终身负责制。严格执行中央关于干部工作生活方面的待遇规定，构建公务用车和办公用房管理长效机制。完善财政资金预算、拨付、绩效评价、投资评审管理制度，强化财政资金使用监管。完善国有资产监管体制和监管方式，进一步规范国有资产处置、产权变更等程序。强化行政监察和审计监督，严肃查处违法违规案件。加强公务员廉洁从政监督与管理，严肃查处以权谋私、失职渎职等行为，树立清正廉洁的新形象。

　　各位代表，一元复始，万象更新，展望未来，信心满怀。尽管在前进的道路上有困难、有风险，但只要全市上下同心同德、开拓奋进，我们就没有克服不了的困难，就没有战胜不了的风险。我们有理由相信，鄂尔多斯经济社会发展前景广阔、潜力巨大，鄂尔多斯的明天一定会更加美好。让我们在市委的坚强领导下，凝心聚力，共克时艰，扎实做好改革发展稳定各项工作，为建设更具实力、更加美丽、更富活力、更为幸福的鄂尔多斯而努力奋斗！
